# Oenothein B Suppresses Lipopolysaccharide (LPS)-Induced Inflammation in the Mouse Brain

**DOI:** 10.3390/ijms14059767

**Published:** 2013-05-07

**Authors:** Satoshi Okuyama, Nahomi Makihata, Morio Yoshimura, Yoshiaki Amakura, Takashi Yoshida, Mitsunari Nakajima, Yoshiko Furukawa

**Affiliations:** 1 Department of Pharmaceutical Pharmacology, College of Pharmaceutical Sciences, Matsuyama University, 4-2 Bunkyo-cho, Matsuyama, Ehime 790-8578, Japan; E-Mails: sokuyama@cc.matsuyama-u.ac.jp (S.O.); n_makihata@cc.matsuyama-u.ac.jp (N.M.); mnakajim@cc.matsuyama-u.ac.jp (M.N.); 2 Department of Pharmacognosy, College of Pharmaceutical Sciences, Matsuyama University, 4-2 Bunkyo-cho, Matsuyama, Ehime 790-8578, Japan; E-Mails: myoshimu@cc.matsuyama-u.ac.jp (M.Y.); amakura@cc.matsuyama-u.ac.jp (Y.A.); tyoshida@gem.e-catv.ne.jp (T.Y.)

**Keywords:** oenothein B, lipopolysaccharide, depression-like behavior, hippocampus, inflammation, microglia, COX-2, astrocyte

## Abstract

Oenothein B has been recently evaluated for its ability to affect inflammatory responses in peripheral tissues. In this study, we examined its effect on the damage to the central nervous system due to systemic inflammation. For this purpose, ICR mice were injected with an intraperitoneal (*i.p.*) dose of lipopolysaccharide (LPS; 1 mg/kg mouse). When oenothein B was administered per os (*p.o.*), it suppressed (1) LPS-induced abnormal behavior in open field; (2) LPS-induced microglial activation in the hippocampus and striatum; and (3) LPS-induced cyclooxygenase (COX)-2 production in the hippocampus and striatum of these mice. These results suggest that oenothein B had the ability to reduce neuroinflammation in the brain during systemic inflammation.

## 1. Introduction

**Figure 1:**
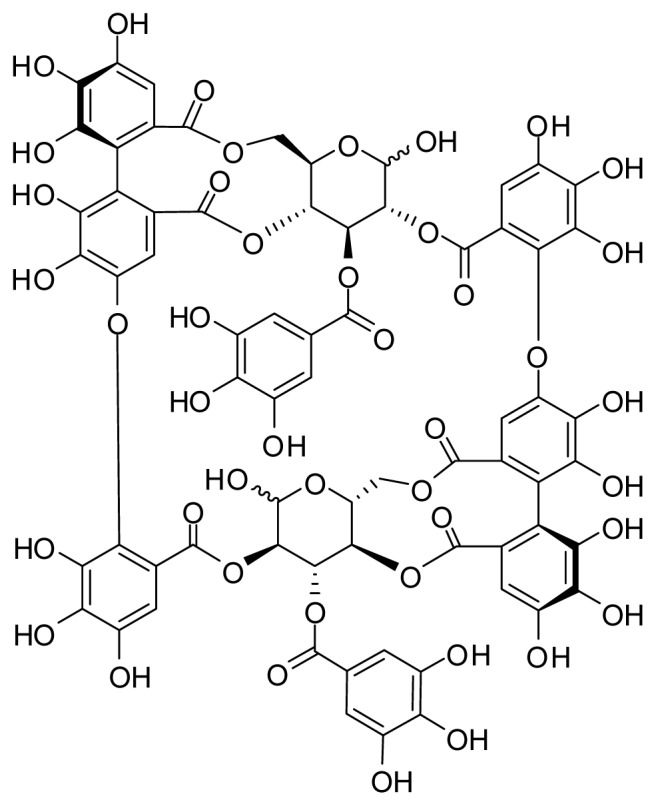
Structure of oenothein B.

Oenothein B (Figure 1), a dimeric macrocyclic ellagitannin, is widely distributed in several medicinal plants belonging to Onagraceae, Lythraceae, and Myrtaceae, including species of *Eucalyptus* [1], *Epilobium* [2], and *Oenothera* [3]. Many plants of these genera have been traditionally used for various medical purposes such as prostate and gastrointestinal disorders, wound healing, and skin stress [4]. The active components responsible for the therapeutic effects of their extracts had not been well defined for a long time, but recent *in vitro* and *in vivo* studies revealed that oenothein B is one of the main biologically active components present in these extracts [5–8]. Detailed *in vitro* examination showed that oenothein B has anti-inflammatory activity against Toll-like receptor (TLR)-stimulated RAW 264.7 macrophages [9]; immunomodulatory activity toward human monocytic THP-1 Blue cells and human leukemia HL-60 cells [6]; and inhibitory activity against prostatic 5α-reductase and aromatase in prostate cancer cell lines PC-3 cells [10]. As part of studies on the activities of oenothein B, we earlier reported that it has anti-tumor activity against MM2 ascites tumors [11] and human oral squamous cell carcinoma [12,13], anti-oxidant activity [1], and an immunomodulatory effect on human dendritic cells [14]. All these results indicated that oenothein B has various potent effects on various peripheral tissues. However, there has been to date no evaluation of the actions of oenothein B in the brain. The objective of this study was thus to ascertain the effects of oenothein B in the brain during systemic inflammation.

As a model animal of systemic inflammation, we used mice that had been intraperitoneally (*i.p.*) administered lipopolysaccharide (LPS), a bacterial endotoxin that interacts with TLR4 receptors on macrophages and elicits a rapid immune response. The LPS-initiated cascade leads to production of a variety of proinflammatory cytokines such as tumor necrosis factor (TNF)-α, interleukin (IL)-1β, and IL-6 [15,16] and to the transcription of the inducible isoform of nitric oxide synthase (iNOS) [17] in the peripheral cavity. These cytokines can cross the blood-brain barrier (BBB), after which they activate resident microglia within the brain [18]; and they also activate endothelial cells and vagal afferents, resulting in the activation of inflammatory responses in the brain [19,20]. On the other hand, a recent report indicated that peripherally injected low doses of LPS directly induce microglial activation in the brain [21]. Anyhow, peripherally injected LPS has the ability to induce immune responses in the brain, resulting in depression-like behavior [22] or mnesic deficits [23,24].

Thus, in this present study we first examined whether oenothein B was effective against LPS-induced abnormal behavior. Finding this to be so, we then examined the effect of oenothein B on the pro-inflammatory responses, such as microglial activation, the expression of cyclooxygenase (COX)-2 and IL-1β, in the hippocampus and striatum.

## 2. Results

### 2.1. Effect of Oenothein B on the Abnormal Behavior Induced by LPS

**Figure 2:**
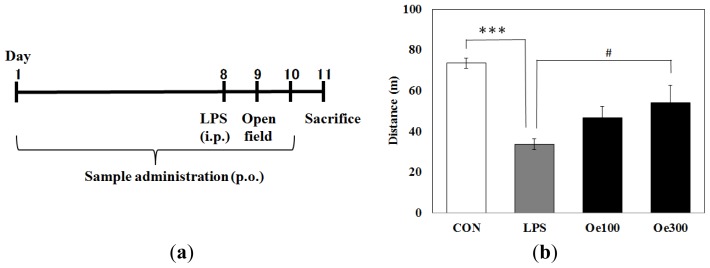
(**a**) Experimental protocol for lipopolysaccharide (LPS)-induced systemic inflammation; (**b**) Effects of LPS (*i.p.*) and it plus oenothein B (*p.o.*) on locomotor activity in the open-field test. Total distance travelled during 10 min was shown. Values are means ± SEM (*n* = 5 for each group). Symbols indicate significant differences as indicated by the brackets: *vs*. control (CON) (*** *p* < 0.001) and *vs*. LPS (^#^*p* < 0.05).

Mice were administered oenothein B per os (*p.o*.) to achieve 100 mg/kg/day (Oe 100 group) or 300 mg/kg/day (Oe 300 group). The control group (CON group) and LPS-treated group (LPS group) were treated with vehicle (distilled water). First we investigated the effect of oenothein B on the LPS-induced abnormal behavior observed in the open-field test. As shown in Figure 2b, the total distance travelled in 10 min for the LPS group was markedly lower (33.8 ± 2.5 m) than that for the CON group (73.6 ± 2.5 m), and there was a significant difference between them (*** *p* < 0.001). This value for the Oe 100 group (46.8 ± 5.6 m) was greater than that for LPS group, but the difference was not significant (*p* = 0.078). However, the higher dose of oenothien B (Oe 300 group) did significantly (^#^*p* < 0.05) increase the locomotive activity over that of the control (54.1 ± 8.6 m).

### 2.2. Effect of Oenothein B on the Microglial Activation Induced by LPS

It has been reported that activated microglia contribute to neurodegenerative diseases by producing various cytotoxic molecules including proinflammatory cytokines [25–27]. So we then stained microglia in the hippocampal regions with anti-ionized calcium-binding adaptor molecule 1 (IBA1) antibody.

**Figure 3:**
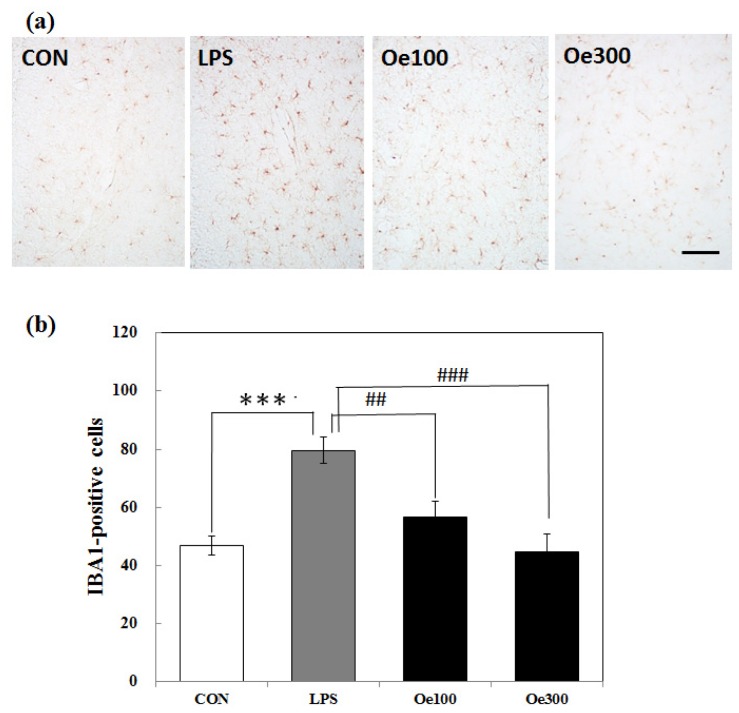
Expression of microglia (immunoreactivity of ionized calcium-binding adaptor molecule 1; IBA1) in the mouse hippocampus. (**a**) Sagittal sections of the hippocampus prepared 3 days after the LPS *i.p.* injection were stained with anti-IBA1 antibody. Scale bar shows 100 μm; (**b**) Quantitative analysis of IBA1-positive cells in the hippocampus by use of Image J software. Values are means ± SEM (*n* = 10~13 for each group). Symbols indicate significant differences as shown by the brackets: *vs*. CON (*** *p* < 0.001) and *vs*. LPS (^###^*p* < 0.001, ^##^*p* < 0.01).

In the CON group, only a few IBA1-positive cells were observed as being in the ramified form (an inactivated form) in the hippocampus (Figure 3a-CON). In the LPS group, the shape of the IBA1-positive cells changed to “ameboid microglia” (an activated form; Figure 3a-LPS) as previously reported [28]; and the number of these cells in the hippocampus was significantly increased (Figure 3b; *** *p* < 0.001). In the Oe 100 and 300 groups, the shape of the IBA1-positive cells indicated the inactive ramified form (Figure 3a-Oe100, Oe300), and the number of these cells was significantly lower than that in the LPS group (Figure 3b; ^##^*p* < 0.01, ^###^*p* < 0.001, respectively).

### 2.3. Effect of Oenothein B on the COX-2 Expression Induced by LPS

**Figure 4:**
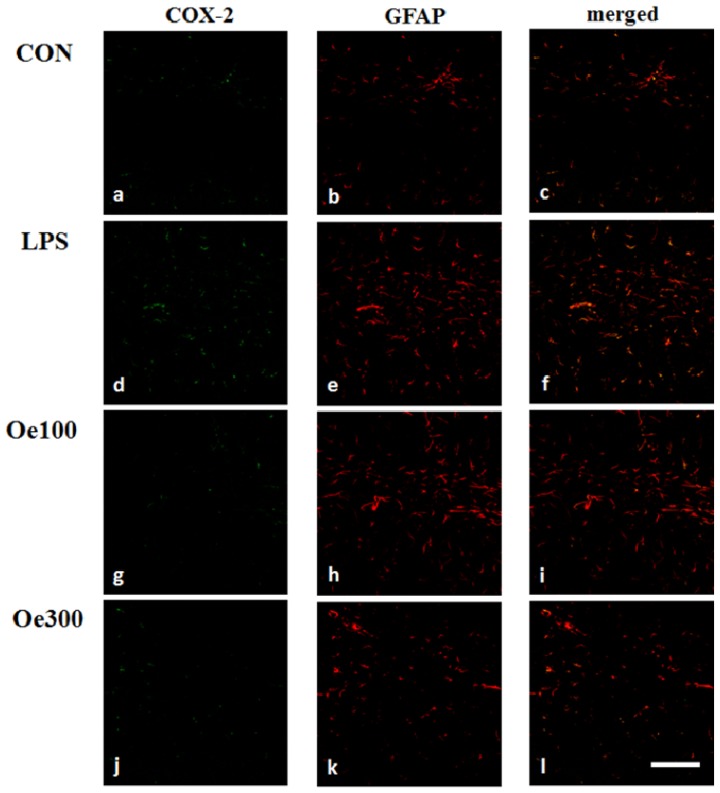
Expression of anti-glial fibrillary acidic protein (GFAP) and cyclooxygenase (COX)-2 in the mouse hippocampus. Sagittal sections prepared 3 days after the LPS *i.p.* injection were stained with specific antibodies, either GFAP (red; **b**,**e**,**h**,**k**) or COX-2 (green; **a**,**d**,**g**,**j**). Merged pictures (**c**,**f**,**i**,**l**) show cells that co-expressed GFAP and COX-2 (yellow cells), meaning that COX-2 was synthesized by activated astrocytes. Scale bar shows 100 μm.

We next examined the pro-inflammatory response in hippocampal regions (CA1 region of Ammon’s horn and dentate gyrus) of the four groups (CON, LPS, Oe 100, and Oe 300) by using COX-2 immunohistochemistry, as COX-2 is well known to be an important enzyme that regulates LPS-induced inflammation [29]. In the CON group, COX-2 immunoreactivity was weakly detected (Figure 4a), but was markedly observed in the LPS group (Figure 4d). In the Oe 100 and 300 groups, the level of this immunoreactivity was reduced to that of the CON group (Figure 4g, j). Recent studies have suggested a crucial importance of astrocytes as well as microglia in inflammatory responses [30]. In these regions, the expression of glial fibrillary acidic protein (GFAP), an astrocytic marker, was increased in the LPS group (Figure 4e) compared with that in the CON group (Figure 4b). The level of GFAP expression in the Oe 100 group (Figure 4h) was similar to that in the LPS group (Figure 4e), but the expression in the Oe 300 group (Figure 4k) was less than that in it (Figure 4e). In all groups, the GFAP-positive cells (astrocytes) were immunopositive for COX-2 (Figure 4c,f,i,l) but the microglia were not (data not shown). These results indicate that activated astrocytes contributed to COX-2 expression after *i.p.* administration of LPS.

### 2.4. Effect of Oenothein B on the IL-1β mRNA Expression Induced by LPS

**Figure 5:**
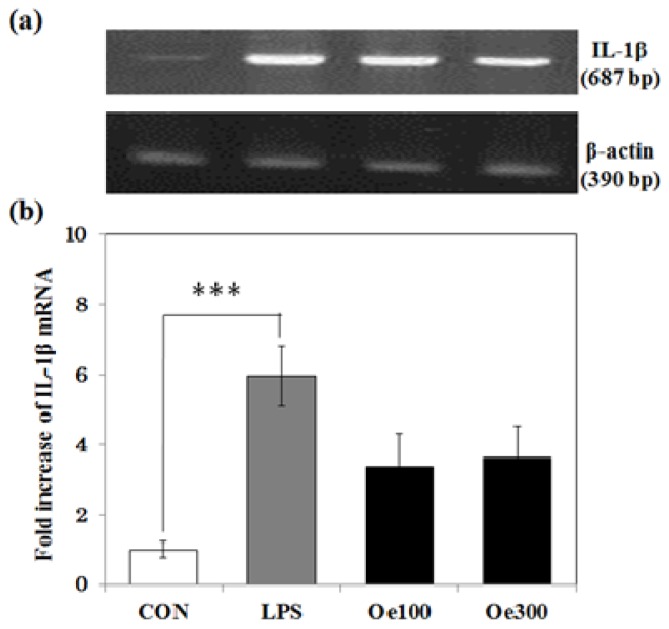
Expression of interleukin (IL)-1β mRNA in the hippocampus. Hippocampal tissues prepared 3 days after *i.p.* injection of mice with LPS were isolated, and RT-PCR analysis was performed with specific primers. (**a**) Densitometric patterns of bands of IL-1β mRNA and actin mRNA; and (**b**) Densitometric quantification of IL-1β mRNA band intensities normalized by the actin mRNA band in the hippocampus. Values are means ± SEM (*n* = 4~5 for each group). Symbols indicate significant difference as indicated by the brackets: *vs.* CON (*** *p* < 0.001).

We next examined the effect of oenothein B on the level of mRNA expression of IL-1β, one of the representative inflammatory cytokines. As shown in Figure 5, LPS treatment significantly increased the level of IL-1β mRNA (*** *p* < 0.001). The administration of oenothein B at either dose level tended to suppress this increase, but the suppression was not statistically significant (*p* = 0.061 for Oe 100 group and *p* = 0.058 for Oe 300 group *vs.* LPS group).

## 3. Discussion

In this study, we addressed the question of whether or not oenothein B has a suppressive effect on inflammatory responses in the brain and on abnormal behavior elicited by systemic inflammation. Here we successfully showed that the *p.o.* administration of oenothein B could suppress both the LPS-induced microglial activation and COX-2 expression in the mouse brain (hippocampus region), as well as the

LPS-induced abnormal behavior seen in the open-field test. To our knowledge, this is the first report to demonstrate that an ellagitannin can suppress inflammatory responses in brain and abnormal behavior. It was previously shown that activation of the immune system in the brain produces psychological and physiological effects, which resemble the characteristics of depression [31]. For investigation of behavior in the open-field test, mice were challenged at 24 h after *i.p.* administration of LPS based on the following previous reports: (1) motor activity in a new cage was decreased at 6 h but not at 24 h; (2) the duration of immobility in the tail suspension test was increased at both 6 h and 24 h; (3) the decreased motor activity and depression-like behavior was confirmed at 24 h in the forced swim test; although sickness behavior was maximal at 6 h and minimal at 24 h after an *i.p.* injection of LPS [32]. In fact, we successfully observed that at 24 h after LPS treatment abnormal behavior was induced and that these symptoms were suppressed by treatment with oenothein B (Figure 2b). We also performed behavioral experiments at 3 days after LPS treatment, but we could not observe any statistically significant difference between the LPS group and CON group (data not shown), probably because the normal locomotor behavior had been restored by 3 days after the LPS injection. On the other hand, we have not yet performed behavioral experiments at an earlier time, such as 6 h. In the next study, we will confirm its effect on the depressive-like behavior by the representative methods, such as forced swimming test and tail suspension test.

For the immunohistochemical and biochemical investigations, we sacrificed the mice at 3 days after LPS treatment. Recent unpublished data of ours showed microglial activation and induction of COX-2 production by astrocytes in the hippocampus and striatum at 1 day after an *i.c.v.* injection of LPS. We predicted that the indirect effect of peripherally injected LPS might require more time than 24 h. In fact, we could observe similar results of our immunohistochemical and biochemical analyses between the mice examined at 3 days after the *i.p.* injection of LPS (Figures 3 and 4) and those observed 1 day after the *i.c.v.* injection of LPS. The LPS-induced COX-2 expression was suppressed by oenothein B, but this suppression showed no statistical significance (Figure 5). As a significant difference might be observed at some earlier time than 3 days, we plan to analyze COX-2 expression at 1 or 2 days after LPS treatment in the near future. A recent report indicated that the expression of COX-2 in neurons and the number of degenerated neurons analyzed by immunohistochemical methods were not significantly changed in the hippocampus after LPS treatment [29]. We also observed no changes in these cells (data not shown).

The most important question to be answered was whether (1) oenothein B or (2) its metabolite(s) passed through the BBB and acted directly in the brain as an anti-inflammatory agent, or (3) oenothein B or (4) its metabolite(s) suppressed the peripheral inflammation, which was followed by the suppression of central inflammation. Oenothein B, hydrophyllic large molecule (MW 1568), might hardly act in passing through the BBB. Recent studies showed that vegetable ellagitannins are metabolized to smaller compounds by intestinal microflora [33,34], and that these metabolites exerted the anti-inflammatory effects on colon fibroblasts [35]. These findings suggested that oenothein B might be absorbed and metabolized to smaller compound(s) by intestinal microflora, and that these intestinal metabolite(s) might suppress the inflammatory responses in the brain. We consider the most plausible explanation for the action of oenothein B to be hypothesis “4”. But there are no adequate data to affirm or negate hypothesis “2” at this time. We will identify the metabolite(s) of oenothien B and investigate the possibility of their permeability through the BBB in the near future.

It has been reported that increased inflammatory responses contribute to brain injury such as stroke and neurodegenerative disorders including Alzheimer’s disease (AD), Parkinson’s disease (PD), *etc.* and that anti-inflammatory drugs have some positive effect on these diseases [36]. Therefore, the results presented here suggest the possibility that both oenothein B and herbs containing it might be potentially beneficial for the treatment of neuroinflammation-related brain diseases.

## 4. Experimental Section

### 4.1. Preparation of Oenothein B

Oenothein B was isolated from the leaves of *Eucalyptus globulus* as described previously [1] with slight modification. In brief, dried leaves of *E. globulus* (1 kg) were extracted with acetone-water (7:3, 10 L), and the filtrate was concentrated by evaporation to 1 L. The concentrate was extracted successively with *n*-hexane (3 L), ethyl acetate (EtOAc, 3 L), and *n*-butanol (BuOH, 3 L); and the final aqueous layer was then chromatographed over Diaion HP-20 (φ 3.0 × 40 cm), with elution using aqueous methanol (MeOH, 10% ↔ 20% ↔ 30%) to MeOH. The 20% MeOH extract was further purified by column chromatography on Sephadex LH-20 (φ 1.1 × 40 cm), with EtOH:MeOH (1:1) for elution to yield oenothein B (1.23 g). HPLC revealed that the purity of oenothein B is more than 95%.

### 4.2. Animals

Six-week-old male ICR strain mice were purchased from Japan SLC (Hamamatsu, Japan). Mice in all groups were kept at 23 ± 1 °C and a 12-h light/dark cycle (light on 8:00–20:00). All animal experiments were carried out in accordance with the Guidelines for Animal Experimentation specified by the Animal Care and Use Committee of Matsuyama University.

### 4.3. Oenothein B Treatment

Oenothein B was dissolved in distilled water. Mice were administered oenothein B per os (*p.o*.) to achieve 100 mg/kg/day (Oe 100 group) or 300 mg/kg/day (Oe 300 group). During the experimental period, the mice were given free access to tap water and food until 08:30 and then deprived of food until the administration time (16:00) of oenothein B or vehicle (0.3 mL-solution).

### 4.4. LPS Treatment

LPS (from *Salmonella enteric* serotype typhimurium) was purchased from Sigma-Aldrich (St. Louis, MO, USA) and dissolved with saline. Immediately after the sample (vehicle or oenothein B) administration at the eighth day, 30 μg LPS in 0.3 mL was *i.p*. administered (1 mg/kg of mouse) as shown in Figure 2a.

### 4.5. Open-Field Test

One day after the LPS injection, locomotive activity was evaluated by using the open-field test. Each mouse was placed in the center of an open field apparatus (W70 × D70 × H50 cm), and free moving behavior was monitored for 10 min. Behavior was analyzed with the ANY-maze Video Tracking System (Stoelting, Wood Dale, IL, USA), which was connected to a CCD camera; and the total distance traveled was analyzed.

### 4.6. Immunohistochemistry

Mice were anesthetized and transcardially perfused with ice-cold PBS. Their brains were then removed and processed for optical microscopy or confocal fluorescence microscopy as previously reported [37]. Sagittal sections at 30 μm were prepared as previously described [37]. For optical microscopy, a rabbit polyclonal antibody against IBA1 (Wako, Osaka, Japan), which is a microglial marker, was used as the primary antibody. The secondary antibody was EnVision-plus system HRP-labeled polymer (anti rabbit; Dako, Glostrup, Denmark). Immunoreactivity was developed and visualized by use of DAB substrate (SK-4100; Vector Laboratories, Burlingame, CA, USA), and quantified by using Image J software (NIH, Bethesda, MD, USA) as described before [28]. For confocal fluorescence microscopy, the primary antibodies used were goat anti-COX-2 (Santa Cruz Biotechnology, Santa Cruz, CA, USA) and mouse anti-GFAP (Sigma-Aldrich, St. Louis, MO, USA); and the secondary antibodies were Alexa Fluor 488-labeled donkey anti-goat IgG (H + L) (Invitrogen, Carlsbad, CA, USA) and Alexa Fluor 568-labeled goat anti-mouse IgG (H + L). The mounting medium used was VECTASHIELD^®^ (Vector Laboratories, Burlingame, CA, USA). Images of the hippocampus were captured with a confocal fluorescence microscopy system (LSM510; Zeiss, Oberkochen, Germany).

### 4.7. RT-PCR Procedures

Total RNA from the hippocampal region of the mice was prepared by use of Isogen (Nippon Gene, Tokyo, Japan), basically composed of guanidine isothiocyanate, and transcribed into cDNA by using a SMART PCR cDNA Synthesis Kit (Clontech, Palo Alto, CA, USA). The synthesized cDNA was amplified by PCR using pairs of primers for IL-1β and actin-β. The numbers of PCR cycles and specific annealing temperature were 38 cycles and 63 °C for IL-1β, 24 cycles and 55 °C for actin-β. The following primer pairs were used: IL-1β, 5′-cttgggctgtccagatgagagcat-3′ and 5′-gaagacacggg ttccatggtgaag-3′; actin-β, 5′-gccgtcttcccctccatcgt-3′ and 5′-cccgtctccggagtccatca-3′. Reaction products (687 bp for IL-1β and 390 bp for actin-β) were electrophoresed on 2% agarose gels containing ethidium bromide. The intensity was measured by using a LAS-3000 imaging system (Fujifilm, Tokyo, Japan).

### 4.8. Statistical Analysis

Data for the individual groups were expressed as means ± SEM. Data were analyzed by one-factor ANOVA followed by Bonferroni’s Multiple Comparison Test (Prism 5; GraphPad Software, La Jolla, CA, USA). Significance is defined as *p* < 0.05.

## 5. Conclusions

Oenothein B, a dimeric macrocyclic ellagitannin, is widely distributed in several medicinal plants. When oenothein B was administered per os (*p.o.*), it suppressed (1) LPS-induced abnormal behavior; (2) LPS-induced microglial activation in the hippocampus and striatum; and (3) LPS-induced COX-2 production in the hippocampus and striatum of these mice. These results suggest that oenothein B had the ability to reduce neuroinflammation in the brain during systemic inflammation.
